# Optimization of Mobile Phase Modifiers for Fast LC-MS-Based Untargeted Metabolomics and Lipidomics

**DOI:** 10.3390/ijms24031987

**Published:** 2023-01-19

**Authors:** Tomas Cajka, Jiri Hricko, Lucie Rudl Kulhava, Michaela Paucova, Michaela Novakova, Ondrej Kuda

**Affiliations:** Institute of Physiology of the Czech Academy of Sciences, Videnska 1083, 14200 Prague, Czech Republic

**Keywords:** metabolomics, lipidomics, optimization, liquid chromatography, mass spectrometry, mobile phase, modifiers, additives, LC-MS

## Abstract

Liquid chromatography-mass spectrometry (LC-MS) is the method of choice for the untargeted profiling of biological samples. A multiplatform LC-MS-based approach is needed to screen polar metabolites and lipids comprehensively. Different mobile phase modifiers were tested to improve the electrospray ionization process during metabolomic and lipidomic profiling. For polar metabolites, hydrophilic interaction LC using a mobile phase with 10 mM ammonium formate/0.125% formic acid provided the best performance for amino acids, biogenic amines, sugars, nucleotides, acylcarnitines, and sugar phosphate, while reversed-phase LC (RPLC) with 0.1% formic acid outperformed for organic acids. For lipids, RPLC using a mobile phase with 10 mM ammonium formate or 10 mM ammonium formate with 0.1% formic acid permitted the high signal intensity of various lipid classes ionized in ESI(+) and robust retention times. For ESI(−), the mobile phase with 10 mM ammonium acetate with 0.1% acetic acid represented a reasonable compromise regarding the signal intensity of the detected lipids and the stability of retention times compared to 10 mM ammonium acetate alone or 0.02% acetic acid. Collectively, we show that untargeted methods should be evaluated not only on the total number of features but also based on common metabolites detected by a specific platform along with the long-term stability of retention times.

## 1. Introduction

Over the last decade, liquid chromatography-mass spectrometry (LC-MS) has become the most frequently used tool for analyzing polar and nonpolar metabolites [1]. Some of them, such as trimethylamine *N*-oxide [2], trimethyllysine [3], phenylacetylglutamine [4], diacetylspermine [5], branched-chain amino acids [6], acylcarnitines [7], or various lipid species [8,9], were reported as biomarkers of various diseases (see also the reviews in [10,11]). However, the true breadth of a metabolome or lipidome cannot be captured by a single instrumental platform because biological samples (e.g., plasma, serum, tissues, and cells) could contain metabolites spanning over 40 orders of magnitude on the predicted octanol/water partition coefficient (XlogP) scale and ranging from femtomolar to millimolar concentrations [12]. Hence, in developing methods for metabolomics and lipidomic analyses, the main task is to cover metabolites using as few platforms as possible while maintaining the requisite precision, accuracy, and linear range for the metabolite classes detected with the chosen platforms. Luckily, various LC-MS modes are available to separate small molecules effectively, including different stationary phase chemistries, compositions of mobile phase solvents, and mobile phase modifiers [1].

In metabolomics, reversed-phase liquid chromatography (RPLC) and hydrophilic interaction chromatography (HILIC) are typical LC-based separation methods [13]. RPLC is used to separate apolar to medium-polar metabolites, and HILIC is used to separate highly-polar to medium-polar metabolites with a 15-50 min analysis time [14]. In lipidomics, in addition to RPLC and HILIC [15], normal phase liquid chromatography (NPLC) and supercritical fluid chromatography (SFC) are also options for lipid separation [16]. Usually, the analysis time ranges between 15 and 60 min [17]. In addition, for large-scale metabolomics and lipidomics studies, fast LC-MS methods (<10 min) are attractive to many researchers because the analysis of over a hundred samples per day is feasible [18].

In this context, we systematically investigated the performance of RPLC and HILIC columns under different chromatographic conditions during untargeted metabolomics and lipidomics using different biological matrices. As a result, we aimed to increase the metabolome and lipidome coverage and achieve fast and reproducible LC separation for each investigated platform.

## 2. Results and Discussion

### 2.1. Untargeted Metabolomics

For untargeted metabolomics, we considered HILIC as a method of choice for evaluation due to their advantages, such as the capability to retain highly polar or ionic metabolites and their use of high organic content mobile phases, more compatible with electrospray ionization-mass spectrometry (ESI-MS) compared to RPLC [14]. To this end, we used an ACQUITY UPLC BEH Amide column (50 mm × 2.1 mm i.d.; 1.7 μm particle size) coupled to an ACQUITY UPLC BEH Amide VanGuard pre-column (5 mm × 2.1 mm i.d.; 1.7 μm particle size) and compared its performance using the same mobile phase solvent composition and with seven different mobile phase modifiers. Specifically, we tested modifiers providing an acidic pH (10 mM ammonium formate with 0.125% formic acid; 0.1% formic acid), a neutral pH (10 ammonium formate; 10 ammonium acetate), and a basic pH (10 mM ammonium acetate with 0.01% ammonium hydroxide; 10 mM ammonium bicarbonate; 0.001% ammonium hydroxide). For an initial evaluation, the sequence consisted of the following injections for the ESI(+) and ESI(−) modes: 10× solvent injections to equilibrate a particular platform, 2× method blanks, 10× mixture of polar standards, 10× serum extracts, and 3× cell extracts. Before testing the next mobile phase modifier combination, the column was rinsed for 30 min with a water/acetonitrile gradient to remove the residues of previous mobile phase modifiers.

**Figure 1:**
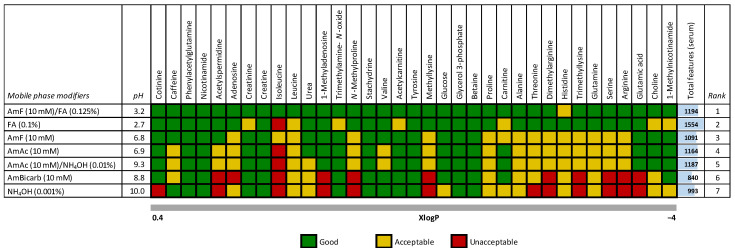
Ranking of 35 polar compounds (endogenous metabolites and exposome compounds) in human serum separated on an ACQUITY UPLC BEH Amide column (50 mm × 2.1 mm i.d.; 1.7 μm particle size) coupled to an ACQUITY UPLC BEH Amide VanGuard pre-column (5 mm × 2.1 mm i.d.; 1.7 μm particle size) under seven mobile phase modifiers in ESI(+). The analytes are sorted according to their predicted octanol/water partition coefficients (XlogP) obtained from the human metabolome database (hmdb.ca); the pH values are reported for the water solution (mobile phase A). Legend: AmF/FA, 10 mM ammonium formate and 0.125% formic acid; FA, 0.1% formic acid; AmF, 10 mM ammonium formate; AmAc, 10 mM ammonium acetate; AmAc/NH_4_OH, 10 mM ammonium acetate and 0.01% ammonium hydroxide; AmBicarb, 10 mM ammonium bicarbonate; NH_4_OH, 0.001% ammonium hydroxide.

First, we evaluated the column performance based on the total number of molecular features in human serum after blank feature subtraction. Here, 0.1% formic acid outperformed other tested modifiers (Figure 1). However, a deeper examination of 35 polar metabolites and exposome compounds (Table S1) typically detected in human serum or plasma using scoring, including the retention time (i.e., compound retention), peak height intensity, and peak width, and the ability to separate leucine/isoleucine isomers, this mobile phase modifier provided sub-optimal results (Figure 1).

**Figure 2:**
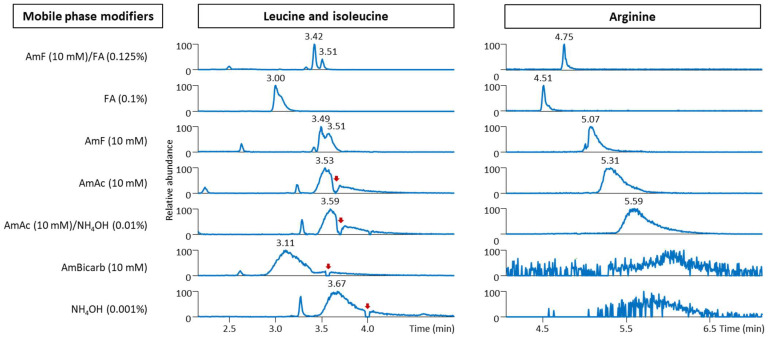
Separation of leucine (*m*/*z* 132.102), isoleucine (*m*/*z* 132.102), and arginine (*m*/*z* 175.120) in human serum extracts using an ACQUITY UPLC BEH Amide column (50 mm × 2.1 mm i.d.; 1.7 μm particle size) coupled to an ACQUITY UPLC BEH Amide VanGuard pre-column (5 mm × 2.1 mm i.d.; 1.7 μm particle size) under seven different mobile phase modifiers. The data were acquired in ESI(+). The red arrows indicate a drop in the signal due to the coelution of highly abundant metabolites or mobile phase impurities. Legend: see Figure 1.

For instance, the leucine/isoleucine isomers were completely separated only when using 10 mM ammonium formate with 0.125% formic acid as the modifiers (Figure 2). This observation is not entirely in line with the results published by Hosseinkhani et al., who reported the coelution of these two isomers on an ACQUITY UPLC BEH Amide column (100 mm × 2.1 mm i.d.; 1.7 μm particle size) [19]. Apparently, 5 mM ammonium formate with the addition of formic acid (pH 3) and an LC gradient initiated at 90% acetonitrile/10% water compared to 95% acetonitrile/5% water used in our study might explain these differences. On the other hand, separating these isomers was feasible using this column with 10 mM ammonium acetate with 0.1% formic acid (pH 3.4) [20]. Using 10 mM ammonium formate with 0.125% formic acid as mobile phase modifiers with a long ACQUITY UPLC BEH Amide column (150 mm × 2.1 mm i.d.; 1.7 μm particle size) was also reported for the baseline separation of these isomers [21,22]. In addition, neutral and basic pH mobile phases often provided broader chromatographic peaks than acidic mobile phases, as shown in Figure 2 for the amino acid arginine. Overall, HILIC in ESI(+) enabled the separation of different classes of polar metabolites, such as amino acids, biogenic amines, sugars, nucleotides, and acylcarnitines. With an optimized gradient, an 8.5 min injection-to-injection run time was feasible.

**Figure 3:**
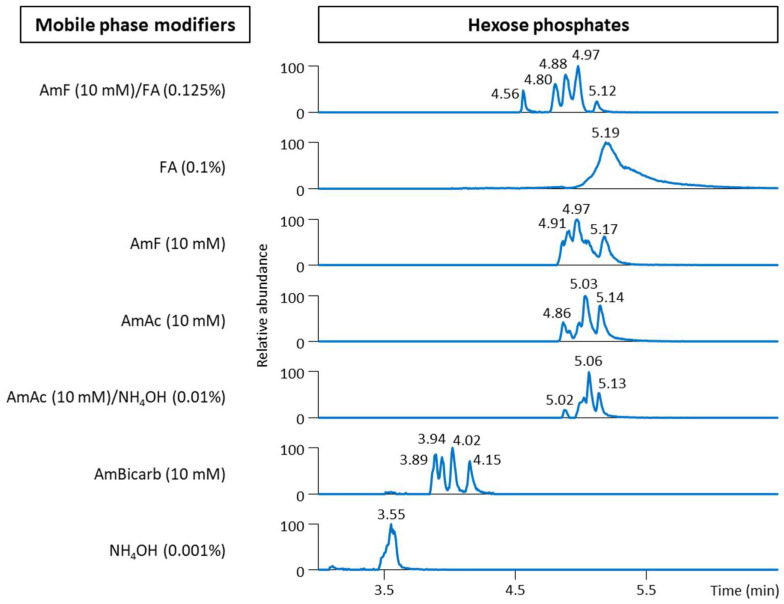
Separation of hexose phosphates (*m*/*z* 259.022) in the cell extracts on an ACQUITY UPLC BEH Amide column (50 mm × 2.1 mm i.d.; 1.7 μm particle size) coupled to an ACQUITY UPLC BEH Amide VanGuard pre-column (5 mm × 2.1 mm i.d.; 1.7 μm particle size) using a mobile phase with seven mobile phase modifiers in ESI(−). The peaks were annotated using authentic standards, including fructose 6-phosphate (4.8 min), glucose 1-phosphate/mannose 6-phosphate/galactose 6-phosphate (4.88 min; individual standards were not separated under the conditions), and glucose 6-phosphate (4.97 min) under the conditions of 10 mM ammonium formate and 0.125% formic acid used as the mobile phase modifiers. Legend: see Figure 1.

None of the evaluated mobile phase modifiers for organic acids evaluated in ESI(−) (Table S2) provided optimal separation. However, the mobile phase with 10 mM ammonium formate and 0.125% formic acid permitted the separation of hexose phosphates in ESI(−). Figure 3 shows that five distinct peaks were observed in cell extracts for hexose phosphates (*m*/*z* 259.022), also confirmed with the MS/MS spectra with class-specific fragments (*m*/*z* 78.959, 96.968, 138.981). When injecting the available authentic standards, we annotated based on the retention time of three of these species.

This fast LC-MS-based untargeted metabolomics method was also tuned regarding MS1 and MS/MS data acquisition. We applied critical rules to increase the number of MS/MS spectra acquired during the data-dependent acquisition (DDA) for metabolite annotation [23]. Among them, the optimal number of MS/MS scans per cycle, the proper threshold for the selection of precursor ion selection, the use of an exclusion list, filters for precursor selection (e.g., isotope exclusion function and charge state), and performing DDA on all measured samples were used to ensure optimal performance [18]. The simultaneous acquisition of MS1 spectra (at 35,000 FWHM resolving power) and three data-dependent MS/MS scans (at 17,500 FWHM resolving power) provided 10–15 data points per chromatographic peak and MS/MS spectra for metabolite annotation.

We further evaluated this HILIC column for intra-batch repeatability using the retention time stability of the representative polar compounds in the plasma extracts. Of the 67 tested compounds (Table S3), all exhibited excellent retention time stability with a relative standard deviation (RSD) of <0.7% (a median RSD of 0.14%, corresponding to a <2 s maximum retention time shift) within 200 injections of plasma extracts.

**Figure 4:**
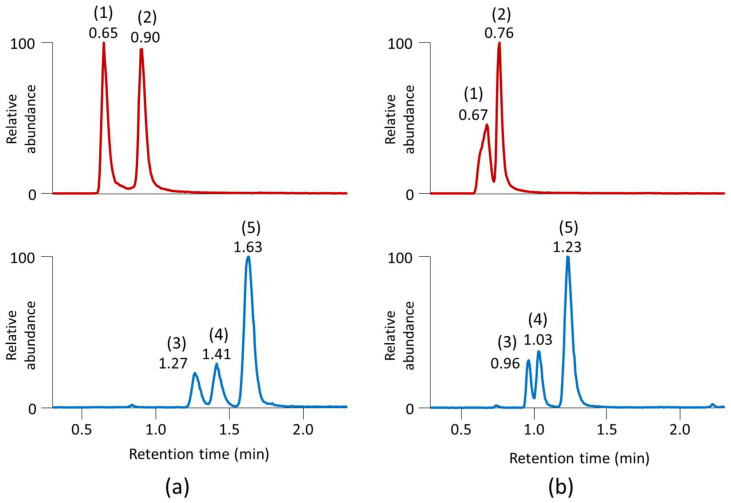
Separation of the (1) isocitrate, (2) citrate (*m*/*z* 191.019), (3) 3-hydroxybutyrate, (4) 3-hydroxyisobutyrate, and (5) 2-hydroxybutyrate (*m*/*z* 103.040) isomers using an ACQUITY UPLC HSS T3 column (50 mm × 2.1 mm i.d.; 1.8 μm particle size) coupled to an ACQUITY UPLC HSS T3 VanGuard pre-column (5 mm × 2.1 mm i.d.; 1.8 μm particle size). (**a**) Gradient water/0.2% formic acid and methanol/0.1% formic acid; (**b**) gradient water/0.2% formic acid and acetonitrile/0.1% formic acid.

Because an ACQUITY UPLC BEH Amide column did not provide sufficient separation of the organic acids in ESI(−) under any of the seven mobile phase modifiers investigated, we focused on RPLC separation. To this end, we used an ACQUITY UPLC HSS T3 column (50 mm × 2.1 mm i.d.; 1.8 μm particle size) coupled to an ACQUITY UPLC HSS T3 VanGuard pre-column (5 mm × 2.1 mm i.d.; 1.8 μm particle size). This column is designed to retain polar compounds and operate at a high aqueous mobile phase [24]. The column efficiency was evaluated by separating a panel of 21 organic acids, such as fumarate, lactate, malate, pyruvate, succinate, *cis*- and *trans*-aconitate, α-ketoglutarate, citrate/isocitrate, 3-hydroxybutyrate/3-hydroxyisobutyrate/2-hydroxybutyrate isomers, and others (Table S2). Interestingly, acidified water and methanol as mobile phases performed better than acidified water and acetonitrile during the gradient separation (Figure 4). With an optimized gradient, a 5.5 min injection-to-injection run time was feasible for separating all the tested organic acids together with the simultaneous acquisition of the MS1 and MS/MS spectra with three data-dependent scans. Again, we evaluated this RPLC column for intra-batch repeatability using the retention time stability. Of the 42 tested compounds (Table S4), all showed an RSD of < 0.9% (a median RSD of 0.5%, corresponding to a <1.5 s retention time shift) within 200 injections of plasma extracts.

### 2.2. Untargeted Lipidomics

RPLC-MS is the most frequently used LC-MS technique in untargeted lipidomics [17]. Different modifiers, such as ammonium formate or acetate alone or in conjunction with formic or acetic acid, were reported in an RPLC-MS analysis of simple and complex lipids [25,26,27,28]. In 2016, Cajka and Fiehn [29] first systematically compared these modifiers with respect to their effects on improving or hampering the electrospray ionization efficiency of specific lipid classes. Using 164 representative lipids covering 11 lipid classes in plasma, they reported that optimal coverage was obtained using 10 mM ammonium formate in ESI(+) and 10 mM ammonium acetate in ESI(−). In follow-up studies, Monnin et al. [30] and Creydl and Fisher [31] pointed out that the use of 0.02% acetic acid improved the signal of lipids in ESI(−) even further compared with ammonium acetate [29].

For our extended evaluation, we used six different mobile phase modifiers, specifically, (i) 10 mM ammonium formate; (ii) 10 mM ammonium formate with 0.1% formic acid; (iii) 10 mM ammonium acetate with 0.1% formic acid; (iv) 10 mM ammonium acetate; (v) 10 mM ammonium acetate with 0.1% acetic acid; and (vi) 0.02% acetic acid. All the mobile phase modifiers were evaluated in the ESI(+) and ESI(−) modes except for 0.02% acetic acid, in which case the ESI(−) data are shown only. For an initial evaluation, the sequence consisted of the following injections for the ESI(+) and ESI(−) modes: 10× solvent injections to equilibrate a particular platform, 2× method blanks, 6× standard lipid mixtures, 6× serum extracts, 3× liver extracts, and 3× tea extracts. Before testing the next mobile phase modifier combination, the column was rinsed for 30 min with a water/acetonitrile gradient to remove the residues of previous mobile phase modifiers. Three different biological matrices were chosen to ensure the annotation of diverse lipid classes.

All samples were separated using a short microbore column ACQUITY UPLC BEH C18 column (50 mm × 2.1 mm i.d.; 1.7 μm particle size) coupled to an ACQUITY UPLC BEH C18 VanGuard pre-column (5 mm × 2.1 mm i.d.; 1.7 μm particle size) because of its frequent use in metabolomics and lipidomics studies [1,17]. For the LC elution, we used mobile phases that were frequently reported in lipidomics studies, specifically (A) acetonitrile:water (60:40) and (B) isopropanol:acetonitrile:water (90:10:0.1) [1,17]. Using an optimized gradient and in combination with C18-modified particles for the LC columns, these solvent combinations permitted the effective elution and separation of complex lipid mixtures within less than 8 min. Similarly to untargeted metabolomics, optimal conditions were obtained for the simultaneous data acquisition of MS1 spectra and three data-dependent MS/MS scans during fast LC-MS untargeted lipidomics methods.

**Figure 5:**
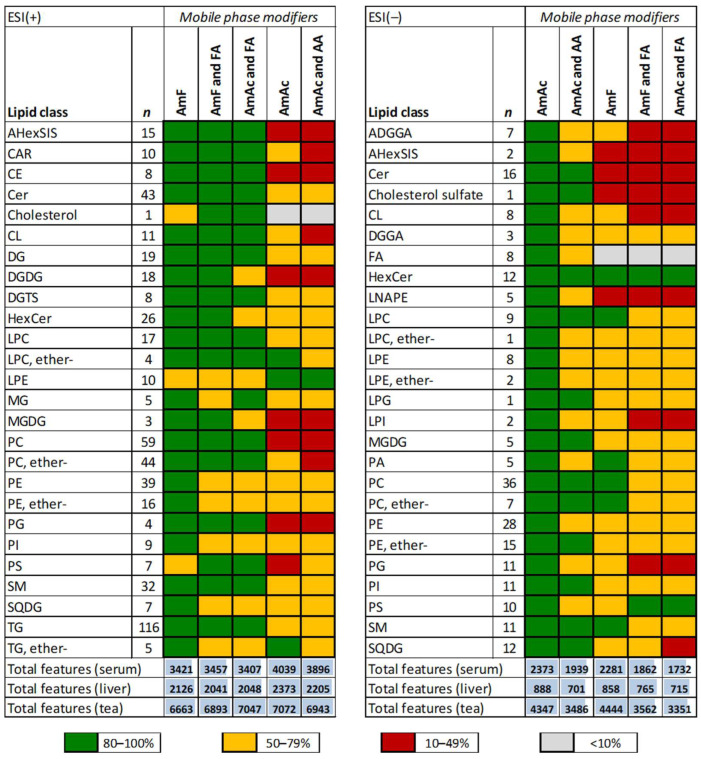
The effect of mobile phase modifiers on the ionization efficiency of lipids in the serum, liver, and tea extracts separated using an ACQUITY UPLC BEH C18 column (50 mm × 2.1 mm i.d.; 1.7 μm particle size) coupled to an ACQUITY UPLC BEH C18 VanGuard pre-column (5 mm × 2.1 mm i.d.; 1.7 μm particle size). The percentages were calculated to the highest peak intensity for each lipid across all samples. For each lipid class, an overall evaluation is marked as excellent (80–100%), sufficient (50–79%), moderate (10–49%), and poor (<10%). Legend: AmAc and AA, 10 mM ammonium acetate and 0.1% acetic acid; AmF and FA, 10 mM ammonium formate and 0.1% formic acid; AmAc, 10 mM ammonium acetate; AmF, 10 mM ammonium formate; AmAc and FA, 10 mM ammonium acetate and 0.1% formic acid. The percentage for each lipid class is found in Table S5. For the lipid abbreviations, see [32]. Examples of extracted ion chromatograms for selected lipids are shown in Figure S1.

An initial evaluation based on the total number of metabolite features after blank feature subtraction indicated that 10 mM ammonium acetate in ESI(+) and 0.02% acetic acid in ESI(−) outperformed compared to other mobile phase modifiers (Table S5). However, since many known lipids are detected through untargeted analysis, we also evaluated these modifiers based on the pattern of annotated lipids. To this end, we selected 536 representative lipids covering 26 lipid classes in ESI(+) and 236 lipids covering 26 lipid classes in ESI(−) (Table S6) and compared their average peak heights for each combination of mobile phase modifiers relative to the most intense peaks across all samples. This targeted approach shows which lipid classes ionized better under the different mobile phase modifiers tested (Figure 5). For ESI(+), 10 mM ammonium formate alone performed the best, followed by 10 mM ammonium formate with 0.1% formic acid. These observations are in line with our previous report [29]. For ESI(−), using 0.02% acetic acid provided overall signal improvement for many lipid classes as recently reported [30]. However, we also observed poor signal intensity for lipid classes, including cardiolipins (CL), phosphatidic acid (PA), and phosphatidylserines (PS), compared to the other modifiers evaluated. Considering all modifiers but 0.02% acetic acid, 10 mM ammonium acetate alone provided the best scoring, followed by 10 mM ammonium acetate with 0.1% acetic acid.

During untargeted lipidomics, the total number of molecular features and annotated lipids and the robustness, such as the stability of retention times, should be considered. The last one is important, specifically during large cohort studies, in which retention time drifting could be an issue during data processing. However, no study has yet addressed this issue for different mobile phase modifiers on a large scale for lipidomics; previous reports [29,30,31] usually conducted <6 injections of lipid standards or biological extracts. Thus, for each mobile phase modifier, we conducted a series of 200 injections (altering the positive and negative ion modes) of the extracts prepared from serum, liver, and tea to achieve high sample complexity. For lipid classes such as (lyso)phosphatidylinositols (LPI and PI), (lyso)phosphatidylglycerols (LPG and PG), PS, PA, sulfoquinovosyl diacylglycerols (SQDG), lyso-*N*-acyl-phosphatidylethanolamine (LNAPE), and cholesterol sulfate we observed a systematic drift of retention times toward longer retention times with increasing injection orders when using 0.02% acetic acid. In comparison, 10 mM ammonium acetate behaved oppositely (shorter retention times with increasing injection orders). However, for the main lipid classes, such as (lyso)phosphatidylcholines (LPC and PC), (lyso)phosphatidylethanolamine (LPE and PE), ceramides (Cer), sphingomyelins (SM), diacylglycerols (DG), triacylglycerols (TG), or free fatty acids (FFA) this phenomenon was not observed.

**Figure 6:**
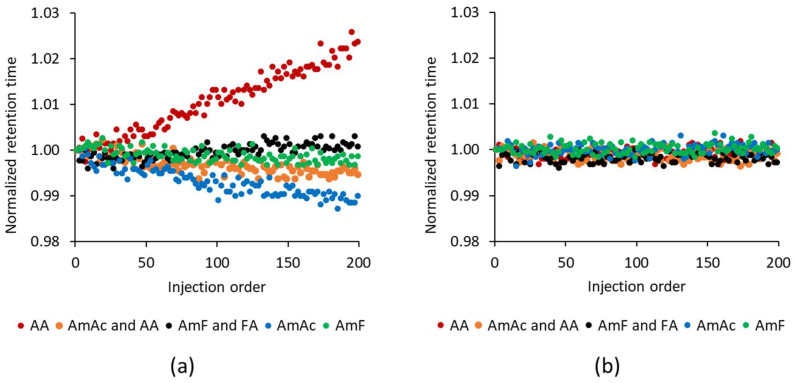
The effect of mobile phase modifiers on the long-term stability (200 injections) of retention times in ESI(−). Examples shown for (**a**) PI 34:3 (PI 16:0_18:3) detected as [M − H]^−^ and (**b**) PC 34:3 (PC 16:0_18:3) detected as [M + CH_3_COO]^−^ or [M + HCOO]^−^. The initial retention time was assigned to 1, and the follow-up retention times were proportionally calculated to provide the normalized retention times. Legend: AA, 0.02% acetic acid; AmAc and AA, 10 mM ammonium acetate and 0.1% acetic acid; AmF and FA, 10 mM ammonium formate and 0.1% formic acid; AmAc, 10 mM ammonium acetate; AmF, 10 mM ammonium formate.

For example, Figure 6 shows two lipid representatives, PI 34:3 (PI 16:0_18:3) and PC 34:3 (PC 16:0_18:3), with opposite retention time drift trends over the sequence with 200 injections. Translating this into absolute retention times using 0.02% acetic acid led to a 4–5 s retention time shift compared to 2 s for 10 mM ammonium acetate alone and 1.5 s for 10 mM ammonium acetate with 0.1% acetic acid during this sequence. The most stable mobile phase modifiers were 10 mM ammonium formate and 10 mM ammonium formate with 0.1% formic acid with retention time shifts of <1 s during 200 injections. Since 10 mM ammonium formate alone and 10 mM ammonium formate with 0.1% formic acid did not provide a high signal intensity for free fatty acids in ESI(−), we concluded that using 10 mM ammonium acetate with 0.1% acetic acid represents a reasonable compromise regarding the signal intensity of the detected lipids and the stability of the retention times.

In general, minimizing retention time drifts is important during the data processing of LC-MS-based metabolomics and lipidomics data sets. Although some software programs [33] are capable of retention time correction using, for instance, internal standards, it is still challenging to perform such corrections if some lipid classes have consistent retention times, while for other ones, systematic drifts may be observed during the sequence.

## 3. Materials and Methods

### 3.1. Materials and Reagents

LC-MS-grade solvents (water, methanol, acetonitrile, and isopropanol), methyl *tert*-butyl ether (MTBE), and LC-MS-grade mobile phase modifiers (formic acid, acetic acid, ammonium hydroxide, ammonium formate, ammonium acetate, and ammonium bicarbonate) were obtained from VWR International, Merck, and J.T.Baker (Prague, Czech Republic). The internal standards for LC-MS were purchased from Merck.

Human serum (S7023-100ML) and plasma (NIST SRM 1950) were obtained from Merck. The mouse liver samples (C57BL/6J) from the GTTAtlas [34] and the cells (3T3-L1) were from the Institute of Physiology of the Czech Academy of Sciences. The tea samples (Earl Grey) were from Bigelow Tea (Fairfield, CT, USA).

### 3.2. Sample Preparation

The metabolomic and lipidomic profiling of the biological samples was conducted using a combined untargeted and targeted workflow for the lipidome, metabolome, and exposome analysis (LIMeX) [34,35]. In principle, the metabolites were extracted using a biphasic solvent system of cold methanol, MTBE, and water [36] with some modifications.

#### 3.2.1. Biofluids (Serum and Plasma)

The serum/plasma samples (25 µL) were mixed with 765 μL of cold methanol/MTBE mixture (165 µL + 600 µL, respectively) containing a mixture of internal standards, and then this mixture was shaken (30 s). Then, 165 µL of 10% methanol with internal standards was added, shaken (30 s), and centrifuged (16,000 rpm, 5 min, 4 °C).

For the lipidomic profiling, 100 µL of the upper organic phase was collected, evaporated, and resuspended using 100 µL methanol with an internal standard (12-[[(cyclohexylamino)carbonyl]amino]-dodecanoic acid, CUDA), shaken (30 s), centrifuged (16,000 rpm, 5 min, 4 °C), and used for LC-MS analysis.

For the metabolomic profiling, 70 µL of the bottom aqueous phase was collected, evaporated, and resuspended in 70 µL of an acetonitrile/water (4:1) mixture with internal standards (CUDA and Val-Tyr-Val), shaken (30 s), centrifuged (16,000 rpm, 5 min, 4 °C), and analyzed using the HILIC metabolomics platform. Another 70 µL aliquot of the bottom aqueous phase was mixed with 210 µL of an isopropanol/acetonitrile (1:1) mixture, shaken (30 s), centrifuged (16,000 rpm, 5 min, 4 °C), and the supernatant was evaporated. The dry extracts were resuspended in 5% methanol/0.2% formic acid with internal standards (CUDA and Val-Tyr-Val), shaken (30 s), centrifuged (16,000 rpm, 5 min, 4 °C), and analyzed using the RPLC metabolomics platform.

#### 3.2.2. Tissues (Liver and Tea)

In total, 20 mg of tissue samples were homogenized (1.5 min) with 275 μL of methanol using a grinder, followed by adding 1 mL of MTBE with an internal standard and shaking (30 s). Then, 275 μL of 10% methanol containing internal standards was added, and the tubes were shaken (1 min) and centrifuged (16,000 rpm, 5 min, 4 °C).

For the lipidomic profiling, 100 µL of the upper organic phase was collected, evaporated, and resuspended using 300 µL of methanol with an internal standard (CUDA), shaken (30 s), centrifuged (16,000 rpm, 5 min, 4 °C), and used for LC-MS analysis.

#### 3.2.3. Cells

A volume of 275 µL of methanol and 275 µL of 10% methanol containing internal standards was put directly on the cells (in a 6-well plate) and then scraped off and transferred to an Eppendorf tube with a milling ball. After homogenization (1 min), 1 mL of MTBE with the internal standard was added, and the tubes were shaken (1 min) and centrifuged (16,000 rpm, 5 min, 4 °C).

For the metabolomic profiling, the procedure was the same as for the biofluids.

### 3.3. LC-MS Conditions

The LC-MS systems consisted of a Vanquish UHPLC system (Thermo Fisher Scientific, Bremen, Germany) coupled to a Q Exactive Plus mass spectrometer (Thermo Fisher Scientific, Bremen, Germany).

#### 3.3.1. Untargeted Metabolomics Using HILIC

The polar metabolites were separated on an ACQUITY UPLC BEH Amide column (50 mm × 2.1 mm i.d.; 1.7 μm particle size) coupled to an ACQUITY UPLC BEH Amide VanGuard pre-column (5 mm × 2.1 mm i.d.; 1.7 μm particle size) (Waters, Milford, MA, USA). The column was maintained at 45 °C at a flow rate of 0.4 mL/min. The mobile phase consisted of (A) water with the following mobile phase modifiers; (i) 0.1% formic acid; (ii) 10 mM ammonium formate and 0.125% formic acid; (iii) 10 mM ammonium formate; (iv) 10 mM ammonium acetate; (v) 10 mM ammonium acetate and 0.01% ammonium hydroxide (pH 9.3); (vi) 10 mM ammonium bicarbonate (pH 8.8); (vii) 0.001% ammonium hydroxide (pH 10.0); and (B) acetonitrile/water (95:5) with the same mobile phase modifiers. In the case of 10 mM ammonium bicarbonate, acetonitrile/water (90:10) was used due to solubility issues. Separation was conducted under the following gradient: 0 min 100% (B); 0–1 min 100% (B); 1–3.9 min from 100% to 70% (B); 3.9–5.1 min from 70% to 30% (B); 5.1–6.4 min from 30% to 100% (B); 6.4–7.5 min 100% (B) +1 min preinjection steps. An injection volume of 1 μL was used. The sample temperature was maintained at 4 °C.

The ESI source and MS parameters were: sheath gas pressure, 50 arbitrary units; aux gas flow, 13 arbitrary units; sweep gas flow, 3 arbitrary units; capillary temperature, 260 °C; aux gas heater temperature, 425 °C. For the untargeted metabolomic profiling, the mass spectrometer was operated under the following conditions: MS1 mass range, *m*/*z* 60–900; MS1 resolving power, 35,000 FWHM (*m*/*z* 200); the number of data-dependent scans per cycle, 3; MS/MS resolving power, 17,500 FWHM (*m*/*z* 200). A spray voltage of 3.6 kV and −2.5 kV for ESI(+) and ESI(−) were used. Normalized collision energies of 20, 30, and 40% were used.

#### 3.3.2. Untargeted Metabolomics Using RPLC

The polar metabolites were also separated on an ACQUITY UPLC HSS T3 column (50 mm × 2.1 mm i.d.; 1.8 μm particle size) coupled to an ACQUITY UPLC HSS T3 VanGuard pre-column (5 mm × 2.1 mm i.d.; 1.8 μm particle size) (Waters, Milford, MA, USA). The column was maintained at 45 °C using a ramped flow rate. The mobile phase consisted of (A) water with 0.2% formic acid, (B) methanol with 0.1% formic acid, or acetonitrile with 0.1% formic acid. Separation was conducted under the following gradient: 0 min 1% (B) 0.3 mL/min; 0–0.5 min 1% (B) 0.3 mL/min; 0.5–2 min from 1% to 60% (B) 0.3 mL/min; 2–2.3 min from 60% to 95% (B) from 0.3 mL/min to 0.5 mL/min; 2.3–3.0 min 95% (B) 0.5 mL/min; 3.0–3.1 min from 95% to 1% (B) 0.5 mL/min; 3.1–4 min 1% (B) 0.5 mL/min; 4–4.1 min 1% (B) from 0.5 mL/min to 0.3 mL/min; 4.1–4.5 min 1% (B) 0.3 mL/min + 1 min preinjection steps. An injection volume of 5 μL was used. The sample temperature was maintained at 4 °C.

The ESI source and MS parameters were: sheath gas pressure, 50 arbitrary units; aux gas flow, 13 arbitrary units; sweep gas flow, 3 arbitrary units; capillary temperature, 260 °C; aux gas heater temperature, 425 °C. For the untargeted metabolomic profiling, the mass spectrometer was operated under the following conditions: MS1 mass range, *m*/*z* 60–900; MS1 resolving power, 35,000 FWHM (*m*/*z* 200); the number of data-dependent scans per cycle, 3; MS/MS resolving power, 17,500 FWHM (*m*/*z* 200). A spray voltage of −2.5 kV for ESI(−) was used. Normalized collision energies of 20, 30, and 40% were used.

#### 3.3.3. Untargeted Lipidomics Using RPLC

The lipids were separated on an ACQUITY UPLC BEH C18 column (50 mm × 2.1 mm i.d.; 1.7 μm particle size) coupled to an ACQUITY UPLC BEH C18 VanGuard pre-column (5 mm × 2.1 mm i.d.; 1.7 μm particle size) (Waters, Milford, MA, USA). The column was maintained at 65 °C at a flow rate of 0.6 mL/min. For the LC-MS analysis, the mobile phase consisted of (A) 60:40 acetonitrile/water with six different mobile phase modifiers: (i) 10 mM ammonium formate; (ii) 10 mM ammonium formate and 0.1% formic acid; (iii) 10 mM ammonium acetate and 0.1% formic acid; (iv) 10 mM ammonium acetate; (v) 10 mM ammonium acetate and 0.1% acetic acid; and (vi) 0.02% acetic acid (B) 90:10:0.1 isopropanol/acetonitrile/water with the same type of mobile phase modifiers. Separation was conducted under the following gradient for LC-ESI(+)-MS: 0 min 15% (B); 0–1 min from 15% to 30% (B); 1–1.3 min from 30% to 48% (B); 1.3–5.5 min from 48% to 82% (B); 5.5–5.8 min from 82% to 99% (B); 5.8–6 min 99% (B); 6–6.1 min from 99% to 15% (B); 6.1–7 min 15% (B) +1 min preinjection steps. For the LC-ESI(−)-MS, the following gradient was used: 0 min 15% (B); 0–1 min from 15% to 30% (B); 1–1.3 min from 30% to 48% (B); 1.3–4.8 min from 48% to 76% (B); 4.8–4.9 min from 76% to 99% (B); 4.9–5.3 min 99% (B); 5.3–5.4 min from 99% to 15% (B); 5.4–6.3 min 15% (B) +1 min preinjection steps. An injection volume of 1–5 μL was used based on the extract type. The sample temperature was maintained at 4 °C.

The source and MS parameters were: sheath gas pressure, 60 arbitrary units; aux gas flow, 25 arbitrary units; sweep gas flow, 2 arbitrary units; capillary temperature, 300 °C; aux gas heater temperature, 370 °C. For the untargeted lipidomic profiling, the mass spectrometer was operated under the following conditions: MS1 mass range, *m*/*z* 200–1700; MS1 resolving power, 35,000 FWHM (*m*/*z* 200); the number of data-dependent scans per cycle, 3; MS/MS resolving power, 17,500 FWHM (*m*/*z* 200). For ESI(+), a spray voltage of 3.6 kV and a normalized collision energy of 20% was used; for ESI(−), a spray voltage of −3.0 kV and normalized collision energies of 10, 20, and 30% were set up.

### 3.4. Data Processing

The LC-MS instrumental files from the metabolomic and lipidomic profiling were processed through MS-DIAL v. 4.92 software [33]. The metabolites were annotated using an in-house retention time–*m*/*z* library and MS/MS libraries available from various sources (NIST20, MoNA, and LipidBlast).

The total number of molecular features was obtained by processing the repeated injections of biological samples and method blanks. For the untargeted metabolomics using HILIC-ESI(+), a series of 10 injections of serum extracts was conducted. For the untargeted lipidomics using RPLC-ESI(+) and RPLC-ESI(−), six injections of serum extracts, three injections of liver extracts, and three injections of tea extracts were performed. First, only the molecular features detected in all replicates for a particular platform and matrix were kept. Then, features passing the criterion “sample maximal signal intensity/blank average signal intensity” >10-fold change (available in MS-DIAL v. 4.92 software) were kept as reliable features for the evaluation of the mobile phase modifiers.

The scoring for the untargeted metabolomics (the HILIC platform) was performed using the (i) retention time (score 0 for a metabolite with a retention time of <1.5 min; score 1 for a metabolite with a retention time of >1.5 min), (ii) peak height intensity (score 1 for a metabolite with the highest peak intensity among all the mobile phase modifiers; for lower peak intensities, proportional decreasing of the score), and (iii) peak width (score −1 for a metabolite with a peak width of >12 s; score 1 for a metabolite with a peak width of <12 s). In addition, for leucine/isoleucine, the ability to resolve these isomers was also considered (score 1 for baseline separation, score 0 for partially resolved peaks, and score −1 for unresolved peaks). The total score for each metabolite was then calculated as a sum of all the scoring values (good score 1.2–3; acceptable score 0–1.2; unacceptable score: <0).

## 4. Conclusions

We evaluated commonly used mobile phase modifiers in analyzing polar metabolites and simple and complex lipids during fast LC-MS. Our findings support that during untargeted metabolomics and lipidomics, the method should not be evaluated just purely based on the total number of features characterized by their retention times and *m*/*z* but should also take into account the common metabolites detected and annotated for particular platforms. In addition, the long-term stability of the retention times should be assessed.

For untargeted metabolomics with an ACQUITY UPLC BEH Amide column, using a mobile phase with 10 mM ammonium formate with 0.125% formic acid provided the best performance for separating amino acids, biogenic amines, sugars, nucleotides, acylcarnitines, and sugar phosphate. However, none of the mobile phase modifiers evaluated performed well for organic acids in ESI(−). For the fast separation and detection of this metabolite class, an ACQUITY UPLC HSS T3 column performed well.

For untargeted lipidomics with an ACQUITY UPLC BEH C18 column, using a mobile phase with 10 mM ammonium formate or 10 mM ammonium formate with 0.1% formic acid permitted a high signal intensity of various lipid classes ionized and detected in ESI(+) along with robust retention times during long runs. For ESI(−), the mobile phase with 10 mM ammonium acetate with 0.1% acetic acid represented a reasonable compromise regarding the signal intensity of the detected lipids and the stability of the retention times compared to 10 mM ammonium acetate alone or 0.02% acetic acid.

Overall, the analysis time between 5.5 and 8.5 min was achieved using optimized LC gradients, permitting fast untargeted LC-MS-based metabolomics and lipidomics methods.

## Data Availability

Not applicable.

## Supplementary Materials

The following supporting information can be downloaded at: https://www.mdpi.com/article/10.3390/ijms24031987/s1.

## References

[1] Cajka T., Fiehn O. (2016). Toward merging untargeted and targeted methods in mass spectrometry-based metabolomics and lipidomics. Anal. Chem..

[2] Wang Z., Klipfell E., Bennett B.J., Koeth R., Levison B.S., Dugar B., Feldstein A.E., Britt E.B., Fu X., Chung Y.M. (2011). Gut flora metabolism of phosphatidylcholine promotes cardiovascular disease. Nature.

[3] Li X.M.S., Wang Z.N., Cajka T., Buffa J.A., Nemet I., Hurd A.G., Gu X.D., Skye S.M., Roberts A.B., Wu Y.P. (2018). Untargeted metabolomics identifies trimethyllysine, a TMAO-producing nutrient precursor, as a predictor of incident cardiovascular disease risk. JCI Insight.

[4] Nemet I., Saha P.P., Gupta N., Zhu W.F., Romano K.A., Skye S.M., Cajka T., Mohan M.L., Li L., Wu Y.P. (2020). A cardiovascular disease-linked gut microbial metabolite acts via adrenergic receptors. Cell.

[5] Wikoff W.R., Hanash S., DeFelice B., Miyamoto S., Barnett M., Zhao Y., Goodman G., Feng Z., Gandara D., Fiehn O. (2015). Diacetylspermine is a novel prediagnostic serum biomarker for non-small-cell lung cancer and has additive performance with pro-surfactant protein B. J. Clin. Oncol..

[6] Mayers J.R., Wu C., Clish C.B., Kraft P., Torrence M.E., Fiske B.P., Yuan C., Bao Y., Townsend M.K., Tworoger S.S. (2014). Elevation of circulating branched-chain amino acids is an early event in human pancreatic adenocarcinoma development. Nat. Med..

[7] Mihalik S.J., Goodpaster B.H., Kelley D.E., Chace D.H., Vockley J., Toledo F.G.S., DeLany J.P. (2010). Increased levels of plasma acylcarnitines in obesity and type 2 diabetes and identification of a marker of glucolipotoxicity. Obesity.

[8] Han X.L., Rozen S., Boyle S.H., Hellegers C., Cheng H., Burke J.R., Welsh-Bohmer K.A., Doraiswamy P.M., Kaddurah-Daouk R. (2011). Metabolomics in early Alzheimer’s disease: Identification of altered plasma sphingolipidome using shotgun lipidomics. PLoS ONE.

[9] Wolrab D., Jirasko R., Cifkova E., Horing M., Mei D., Chocholouskova M., Peterka O., Idkowiak J., Hrnciarova T., Kuchar L. (2022). Lipidomic profiling of human serum enables detection of pancreatic cancer. Nat. Commun..

[10] Tolstikov V., Moser A.J., Sarangarajan R., Narain N.R., Kiebish M.A. (2020). Current status of metabolomic biomarker discovery: Impact of study design and demographic characteristics. Metabolites.

[11] Hornemann T., von Eckardstein A., Binder C.J. (2022). Lipidomics in Biomarker Research. Prevention and Treatment of Atherosclerosis: Improving State-of-the-Art Management and Search for Novel Targets.

[12] Fiehn O., Bloszies C.S. (2018). Using untargeted metabolomics for detecting exposome compounds. Curr. Opin. Toxicol..

[13] Rampler E., El Abiead Y., Schoeny H., Rusz M., Hildebrand F., Fitz V., Koellensperger G. (2021). Recurrent topics in mass spectrometry-based metabolomics and lipidomics-standardization, coverage, and throughput. Anal. Chem..

[14] Tang D.Q., Zou L., Yin X.X., Ong C.N. (2016). HILIC-MS for metabolomics: An attractive and complementary approach to RPLC-MS. Mass Spectrom. Rev..

[15] Medina J., van der Velpen V., Teav T., Guitton Y., Gallart-Ayala H., Ivanisevic J. (2020). Single-step extraction coupled with targeted HILIC-MS/MS approach for comprehensive analysis of human plasma lipidome and polar metabolome. Metabolites.

[16] Lange M., Ni Z.X., Criscuolo A., Fedorova M. (2019). Liquid chromatography techniques in lipidomics research. Chromatographia.

[17] Cajka T., Fiehn O. (2014). Comprehensive analysis of lipids in biological systems by liquid chromatography-mass spectrometry. TrAC-Trend Anal. Chem..

[18] Rakusanova S., Fiehn O., Cajka T. (2023). Toward building mass spectrometry-based metabolomics and lipidomics atlases for biological and clinical research. TrAC-Trend Anal. Chem..

[19] Hosseinkhani F., Huang L.J., Dubbelman A.C., Guled F., Harms A.C., Hankemeier T. (2022). Systematic evaluation of HILIC stationary phases for global metabolomics of human plasma. Metabolites.

[20] Contrepois K., Jiang L., Snyder M. (2015). Optimized analytical procedures for the untargeted metabolomic profiling of human urine and plasma by combining hydrophilic interaction (HILIC) and reverse-phase liquid chromatography (RPLC)-mass spectrometry. Mol. Cell. Proteom..

[21] Ding J., Ji J., Rabow Z., Shen T., Folz J., Brydges C.R., Fan S.L., Lu X.C., Mehta S., Showalter M.R. (2021). A metabolome atlas of the aging mouse brain. Nat. Commun..

[22] Bonini P., Kind T., Tsugawa H., Barupal D.K., Fiehn O. (2020). Retip: Retention time prediction for compound annotation in untargeted metabolomics. Anal. Chem..

[23] Defossez E., Bourquin J., Reuss S., Rasmann S., Glauser G. (2021). Eight key rules for successful data-dependent acquisition in mass spectrometry-based metabolomics. Mass Spectrom. Rev..

[24] Birkler R.I.D., Stottrup N.B., Hermannson S., Nielsen T.T., Gregersen N., Botker H.E., Andreasen M.F., Johannsen M. (2010). A UPLC-MS/MS application for profiling of intermediary energy metabolites in microdialysis samples-A method for high-throughput. J. Pharmaceut. Biomed. Anal..

[25] Bojic L.A., McLaren D.G., Shah V., Previs S.F., Johns D.G., Castro-Perez J.M. (2014). Lipidome of atherosclerotic plaques from hypercholesterolemic rabbits. Int. J. Mol. Sci..

[26] Bird S.S., Marur V.R., Stavrovskaya I.G., Kristal B.S. (2013). Qualitative characterization of the rat liver mitochondrial lipidome using LC–MS profiling and high energy collisional dissociation (HCD) all ion fragmentation. Metabolomics.

[27] Choi J.M., Kim T.E., Cho J.Y., Lee H.J., Jung B.H. (2014). Development of lipidomic platform and phosphatidylcholine retention time index for lipid profiling of rosuvastatin treated human plasma. J. Chromatogr. B.

[28] Gallart-Ayala H., Courant F., Severe S., Antignac J.P., Morio F., Abadie J., Le Bizec B. (2013). Versatile lipid profiling by liquid chromatography-high resolution mass spectrometry using all ion fragmentation and polarity switching. Preliminary application for serum samples phenotyping related to canine mammary cancer. Anal. Chim. Acta.

[29] Cajka T., Fiehn O. (2016). Increasing lipidomic coverage by selecting optimal mobile-phase modifiers in LC–MS of blood plasma. Metabolomics.

[30] Monnin C., Ramrup P., Daigle-Young C., Vuckovic D. (2018). Improving negative liquid chromatography/electrospray ionization mass spectrometry lipidomic analysis of human plasma using acetic acid as a mobile-phase additive. Rapid Commun. Mass. Spectrom..

[31] Creydt M., Fischer M. (2017). Plant metabolomics: Maximizing metabolome coverage by optimizing mobile phase additives for nontargeted mass spectrometry in positive and negative electrospray ionization mode. Anal. Chem..

[32] Tsugawa H., Cajka T., Kind T., Ma Y., Higgins B., Ikeda K., Kanazawa M., VanderGheynst J., Fiehn O., Arita M. (2015). MS-DIAL: Data-independent MS/MS deconvolution for comprehensive metabolome analysis. Nat. Methods.

[33] Tsugawa H., Ikeda K., Takahashi M., Satoh A., Mori Y., Uchino H., Okahashi N., Yamada Y., Tada I., Bonini P. (2020). A lipidome atlas in MS-DIAL 4. Nat. Biotechnol..

[34] Lopes M., Brejchova K., Riecan M., Novakova M., Rossmeisl M., Cajka T., Kuda O. (2021). Metabolomics atlas of oral 13C-glucose tolerance test in mice. Cell Rep..

[35] Sistilli G., Kalendova V., Cajka T., Irodenko I., Bardova K., Oseeva M., Zacek P., Kroupova P., Horakova O., Lackner K. (2021). Krill oil supplementation reduces exacerbated hepatic steatosis induced by thermoneutral housing in mice with diet-induced obesity. Nutrients.

[36] Matyash V., Liebisch G., Kurzchalia T.V., Shevchenko A., Schwudke D. (2008). Lipid extraction by methyl-tert-butyl ether for high-throughput lipidomics. J. Lipid Res..

