# Supplementary material for: Optimization of Mobile Phase Modifiers for Fast LC-MS-Based Untargeted Metabolomics and Lipidomics
Source: Int J Mol Sci. 2023 Jan 19;24(3):1987. doi: 10.3390/ijms24031987 (PMC9916776; doi:10.3390/ijms24031987)

Supplementary Materials

Optimization of Mobile Phase Modifiers for Fast LC-MS-Based Untargeted Metabolomics and Lipidomics

Tomas Cajka *, Jiri Hricko, Lucie Rudl Kulhava, Michaela Paucova, Michaela Novakova and Ondrej Kuda

Institute of Physiology of the Czech Academy of Sciences, Videnska 1083, 14200 Prague, Czech Republic
* Correspondence: tomas.cajka@fgu.cas.cz

Figure S1. Examples of extracted ion chromatograms of different lipid classes. RPLC-ESI(+): PC 36:1, CE 18:2, cholesterol; RPLC-ESI(–): FA 18:2, CL 72:8, PA 34:3, PG 36:6.

Figure S1. Examples of extracted ion chromatograms of different lipid classes. RPLC-ESI(+): PC 36:1, CE 18:2, cholesterol; RPLC-ESI(–): FA 18:2, CL 72:8, PA 34:3, PG 36:6.

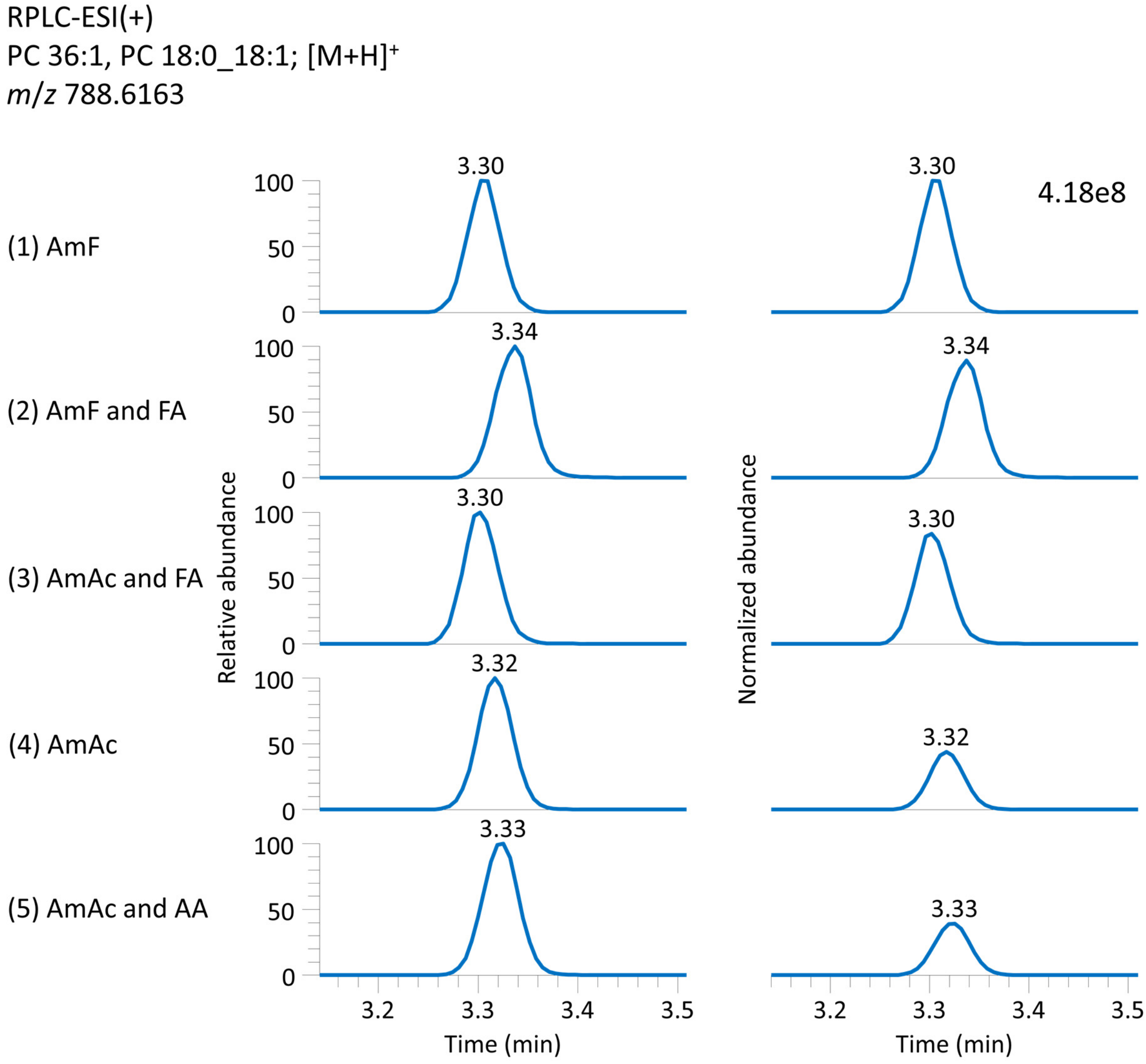

RPLC-ESI(+)
CE 18:2; $[M+NH_4]^+$
m/z 666.6184

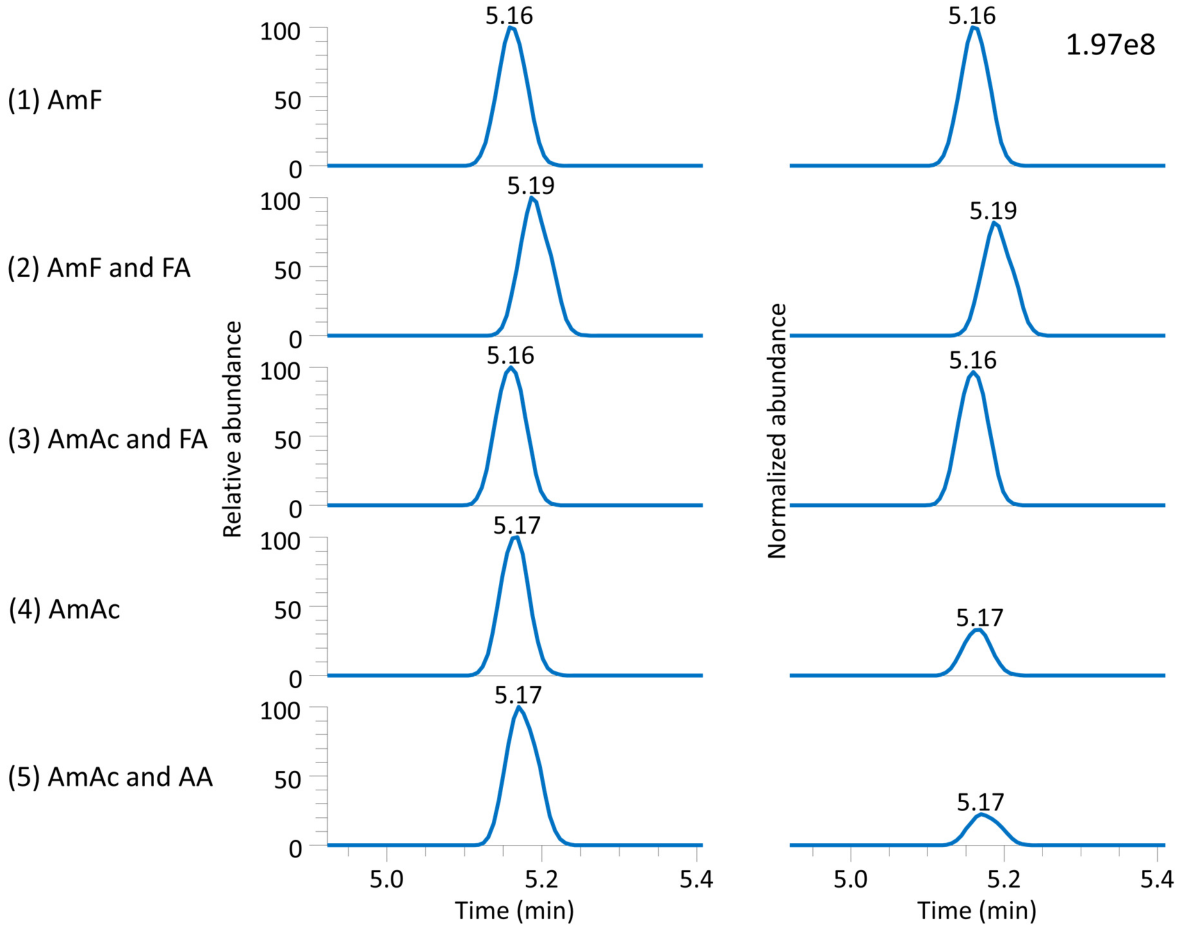

RPLC-ESI(+)
Cholesterol; $[M–H_2O+H]^+$
m/*z* 666.6184

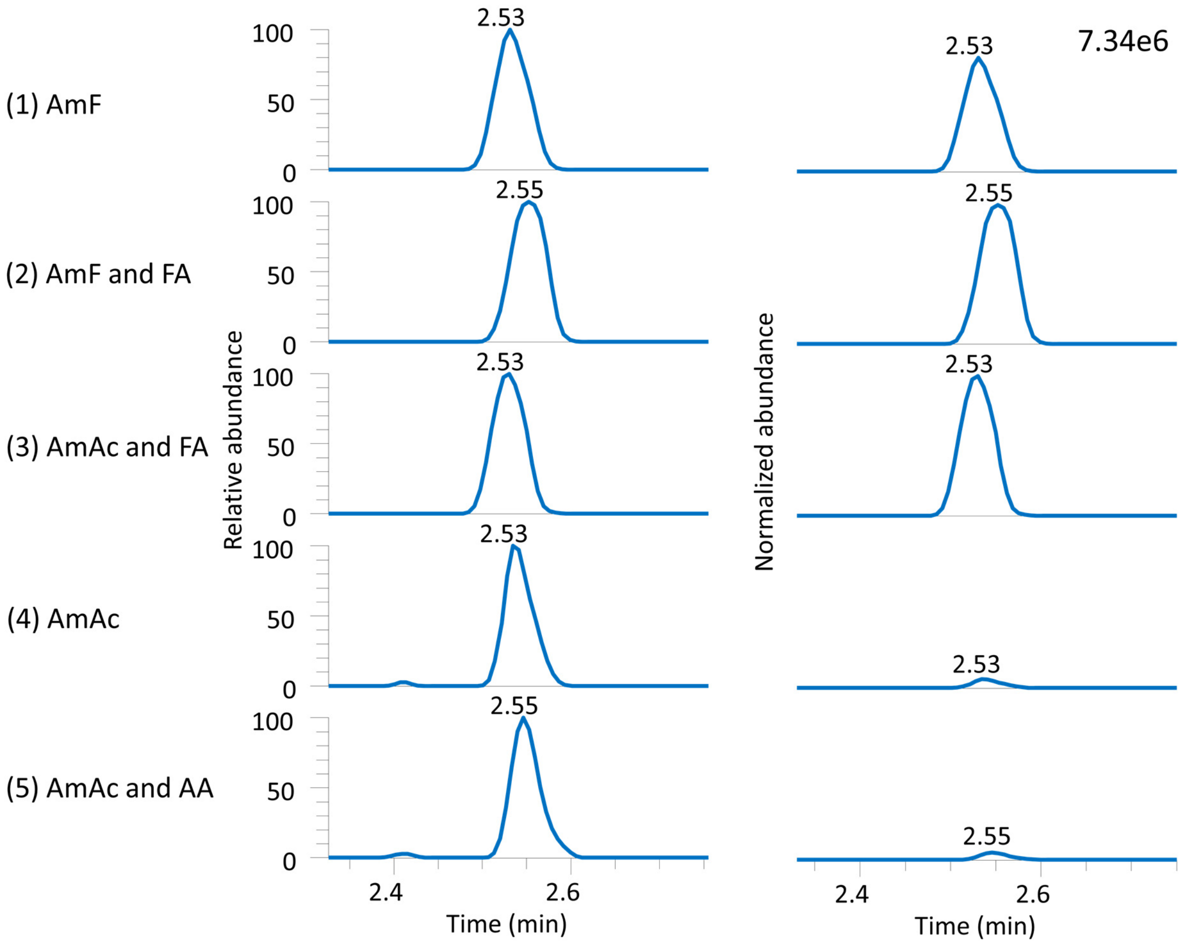

RPLC-ESI(–)
FA 18:2; [M–H]⁻
m/z 279.2329

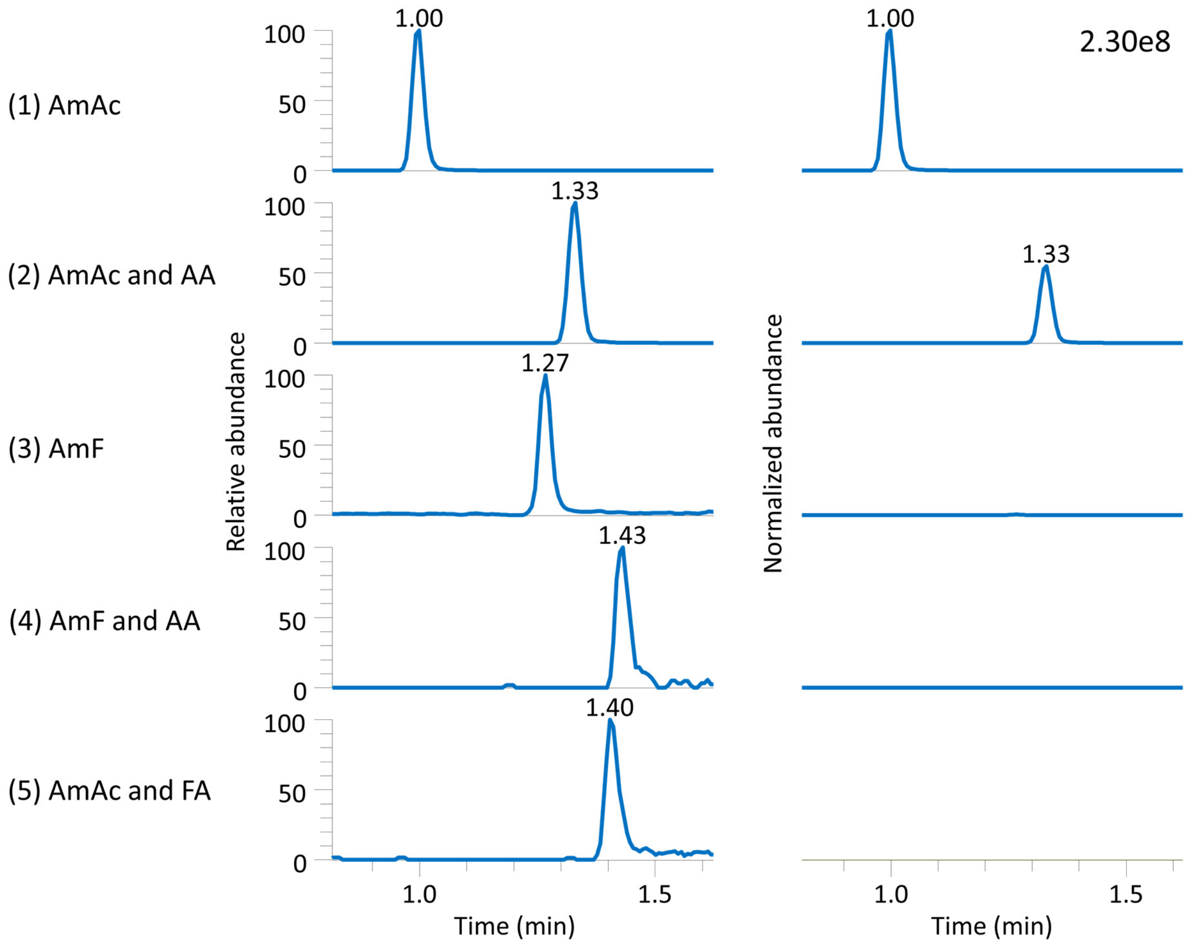

RPLC-ESI(–)
CL 72:8|CL 18:2_18:2_18:2_18:2; [M–H]⁻
m/*z* 1447.9650

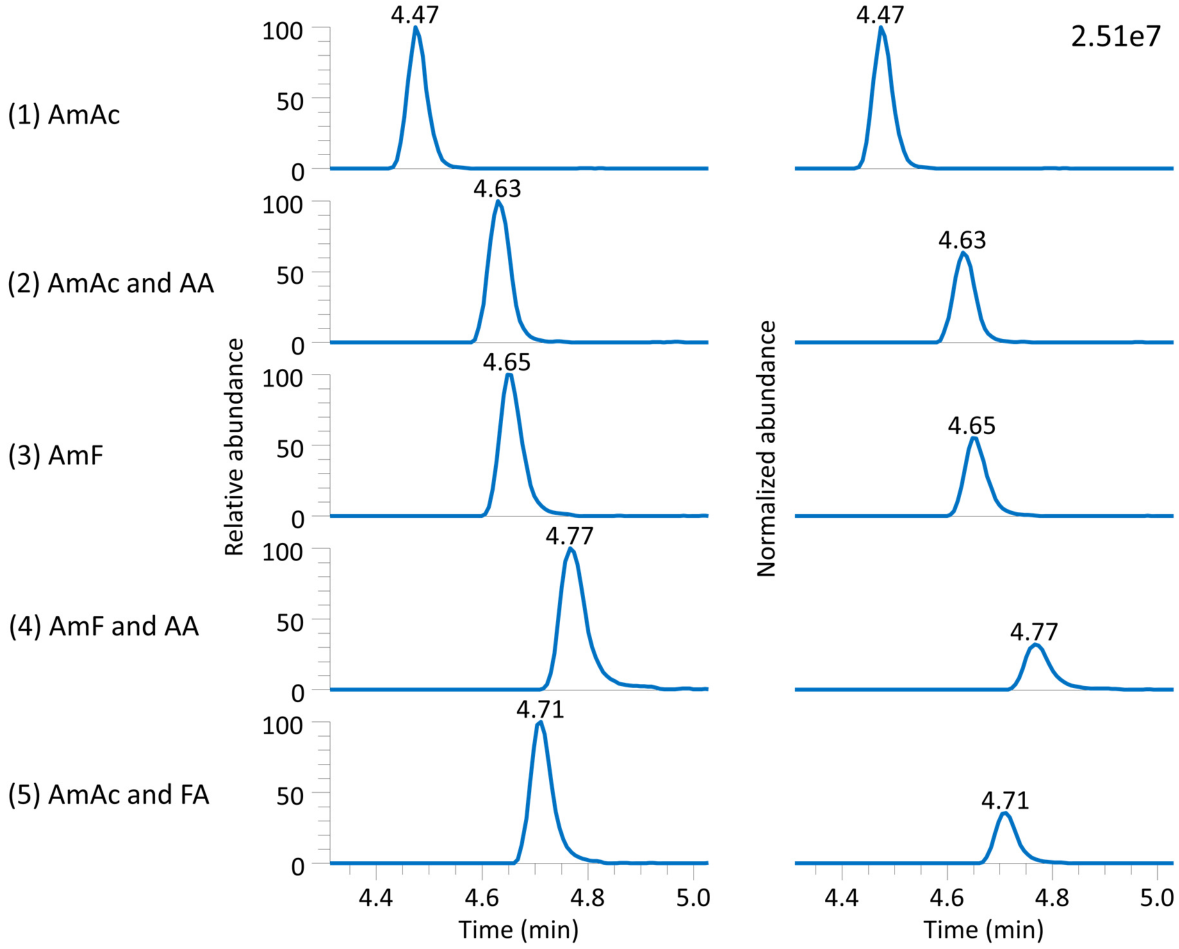

RPLC-ESI(–)
PA 34:3|PA 16:0_18:3; [M–H]⁻
m/*z* 669.4501

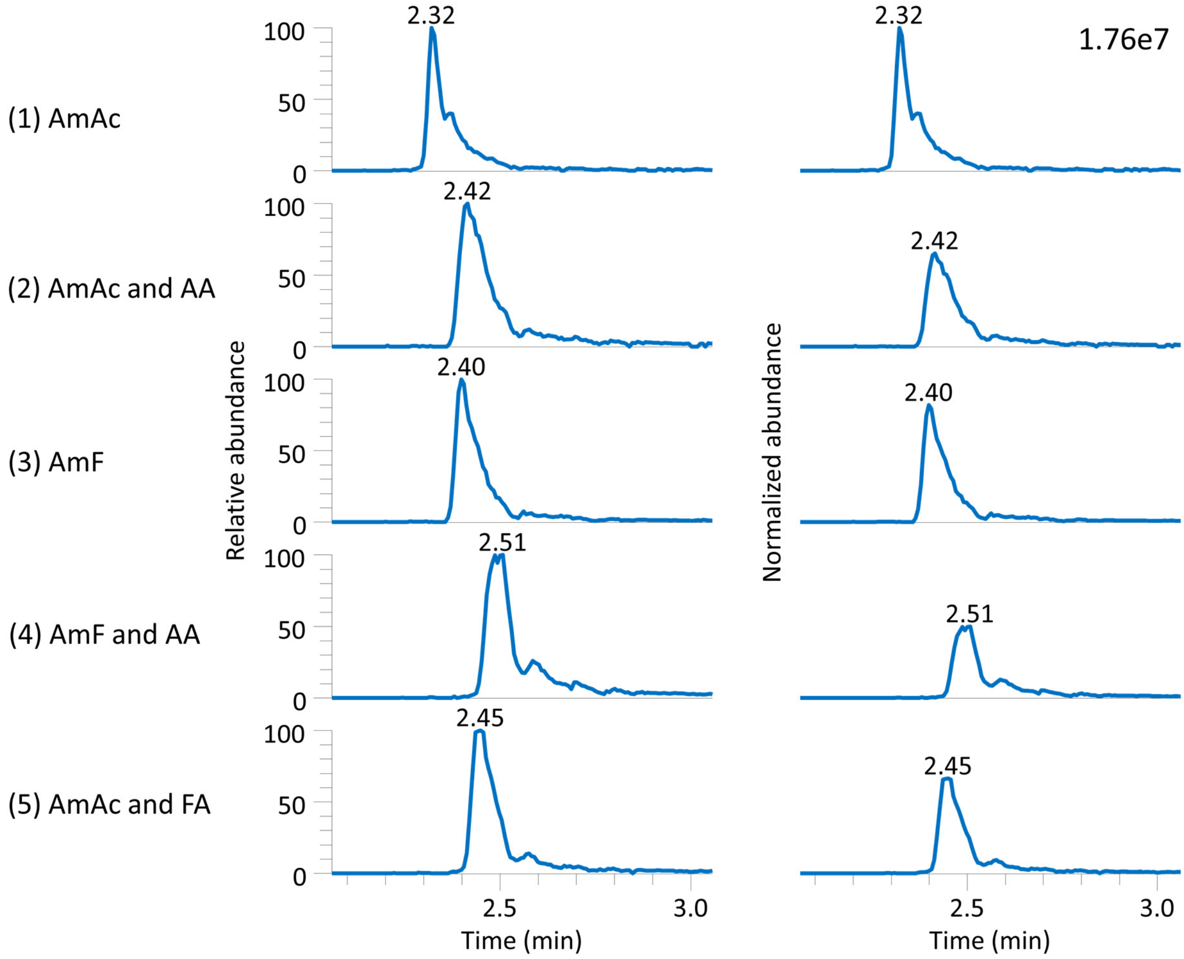

RPLC-ESI(–)
PG 36:6|PG 18:3_18:3; [M–H]⁻
m/z 765.4712

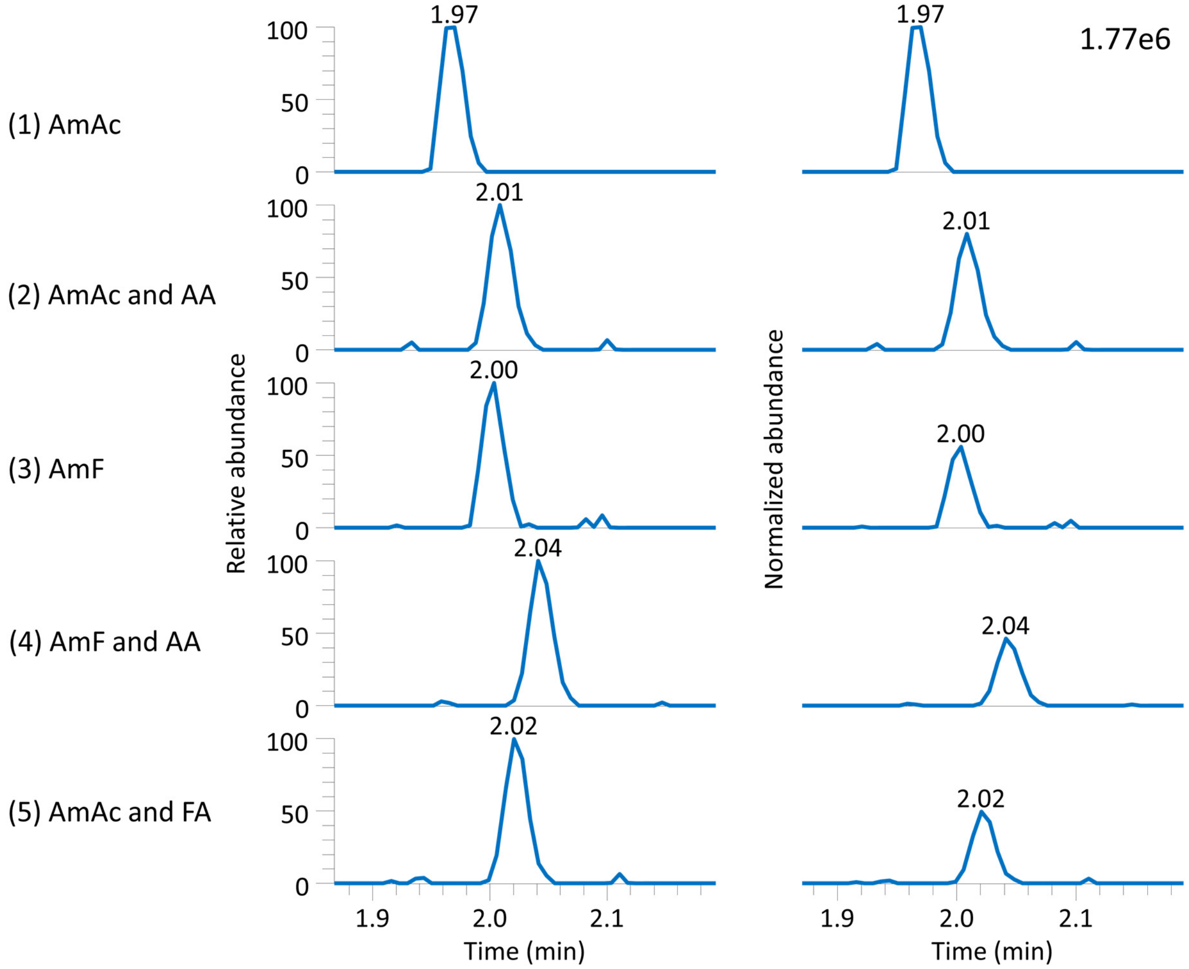

Supplement: Supplementary file 1 [file ijms-24-01987-s001.zip › ijms-2164599_Figure S1.pdf]
